# Association between Urinary Excretion of Cortisol and Markers of Oxidatively Damaged DNA and RNA in Humans

**DOI:** 10.1371/journal.pone.0020795

**Published:** 2011-06-07

**Authors:** Anders Joergensen, Kasper Broedbaek, Allan Weimann, Richard D. Semba, Luigi Ferrucci, Martin B. Joergensen, Henrik E. Poulsen

**Affiliations:** 1 Psychiatric Centre Copenhagen, Copenhagen, Denmark; 2 Laboratory of Clinical Pharmacology Q7642, Rigshospitalet, Copenhagen, Denmark; 3 Department of Clinical Pharmacology, Bispebjerg Hospital, Copenhagen, Denmark; 4 Johns Hopkins University School of Medicine, Baltimore, Maryland, United States of America; 5 Longitudinal Studies Section, Clinical Research Branch, Gerontology Research Center, National Institute on Aging, Baltimore, Maryland, United States of America; 6 Faculty of Health Sciences, University of Copenhagen, Copenhagen, Denmark; University of Valencia, Spain

## Abstract

Chronic psychological stress is associated with accelerated aging, but the underlying biological mechanisms are not known. Prolonged elevations of the stress hormone cortisol is suspected to play a critical role. Through its actions, cortisol may potentially induce oxidatively generated damage to cellular constituents such as DNA and RNA, a phenomenon which has been implicated in aging processes. We investigated the relationship between 24 h excretion of urinary cortisol and markers of oxidatively generated DNA and RNA damage, 8-oxo-7,8-dihydro-2′-deoxyguanosine and 8-oxo-7,8-dihydroguanosine, in a sample of 220 elderly men and women (age 65 – 83 years). We found a robust association between the excretion of cortisol and the oxidation markers (*R^2^* = 0.15, *P*<0.001 for both markers). Individuals in the highest quartile of cortisol excretion had a 57% and 61% higher median excretion of the DNA and RNA oxidation marker, respectively, than individuals in the lowest quartile. The finding adds support to the hypothesis that cortisol-induced damage to DNA/RNA is an explanatory factor in the complex relation between stress, aging and disease.

## Introduction

Modern biomedical research has shed light on the popular notion that psychological stress has a negative influence on health and accelerates aging. Prolonged stress is thought to induce a “wear and tear” syndrome, in which a range of compensatory physiological mechanisms as well as behavioural changes leads to negative health influences [1]. For example, it is well established that chronic stress increases the risk of cardiovascular disease [2]. Stress may also have atrophic effects in distinct areas of the brain [3], induce immunosuppression, and contribute to the progression of some kinds of cancer [4]. Stress-related mental disorders such as depression are associated with an increased non-suicide mortality [5].

The central link between prolonged psychological stress, aging, and disease, is suspected to be chronic elevations of cortisol and other stress hormones. Where the acute cortisol response to stress is necessary for survival, psychological stress associated with prolonged hypercortisolism supposedly leads to a state of stable dysregulation that is detrimental to health over time [1]. However, the underlying molecular mechanisms have yet to be elucidated [6]. One possibility is that the combined effects of cortisol lead to increased *oxidative stress*, in which the mitochondrial production of reactive oxygen species (ROS) exceeds the antioxidant potential, thereby causing damage to other molecules such as lipids, proteins and DNA/RNA. Oxidative stress, in particular the oxidatively generated damage to DNA, has been suggested to be a central mediator of aging [7], [8].

In animal studies, psychological stress and exogenous corticosteroid administration induces increased oxidatively damaged DNA and other measures of oxidative stress [9]–[11]. In one study, oxidatively damaged DNA in rat liver mitochondria increased after chronic administration of corticosterone (the rodent analog of cortisol) in a dose-dependent manner [12]. Oxidatively damaged DNA was increased in clinical studies of occupational stress [13] and in clinical depression [14].

The oxidation of DNA and RNA generates a range of free nucleosides, presumably due to the subsequent excision by repair enzymes. Among these nucleosides, one of the most frequently measured is 8-oxo-7,8-dihydro-2′-deoxyguanosine (8-oxodG), a product of the oxidation of guanine. 8-oxodG is a marker of total oxidatively generated DNA damage, and is also a mutagenic lesion in itself [8]. The compound is excreted and measurable in urine [15].

One previous report found a positive correlation between serum cortisol levels and spot urine sample 8-oxodG concentrations in healthy, middle-aged Japanese workers, but the association did not remain significant in multivariate analysis [13]. Due to the high diurnal variation in cortisol and susceptibility to variation in relation to venipuncture, a 24 h urinary sampling is superior to blood sampling when estimating overall secretion. Furthermore, when measuring nucleic acid excretion in spot urine samples, a correction with urinary creatinine is applied. When using 24 h samples, this potential confounder is avoided.

In this study, we measured markers of both DNA and RNA oxidation (8-oxodG and 8-oxo-7,8-dihydroguanosine (8-oxoGuo), respectively) in 24 h urine samples from a subsample of the InCHIANTI cohort (Invechiarre in Chianti, aging in the Chianti Region). We compared the 24 h 8-oxodG and 8-oxoGuo excretion to the excretion of cortisol determined from the same samples. To our knowledge, the simultaneous determination of urinary markers of oxidatively generated DNA/RNA damage and cortisol from a 24 h urine sample has not previously been performed. Based on the above, we hypothesized a positive association between 24 h urinary cortisol and 8oxodG/8-oxoGuo excretion.

## Results

**Figure 1:**
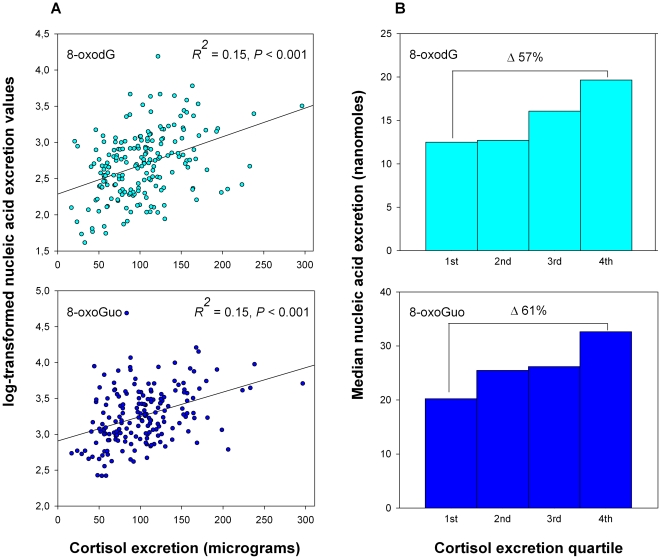
24 h urinary excretion of cortisol vs markers of nucleic acid oxidation. A: Scatterplot of log-transformed 8-oxodG and 8-oxoGuo values vs 24 h cortisol excretion. B: Median nucleic acid excretion vs quartiles of cortisol excretion. Median 8-oxodG and 8-oxoGuo is 57% and 61% percent higher, respectively, in individuals in the highest vs individuals in the lowest cortisol excretion quartile.

Demographic, clinical and biochemical characteristics are shown in Table 1. Linear regression analysis showed significant positive relationships between both 8-oxodG and 8-oxoGuo and cortisol excretion (*R^2^* = 0.15, *P*<0.001 for both markers) (Figure 1A). This result persisted after the adjustment for multiple known or possible confounders of oxidative stress (age, sex, BMI, serum ferritin, blood glucose, insulin, inflammation status, smoking status and previous diagnosis of cancer) in multivariate analysis, in which only serum ferritin (as previously reported [16]) and cortisol were significantly associated with the oxidation markers (Table 2). Due to the specific relation between DNA damage and cancer [8], we ran all analyses without the 22 subjects with a previous diagnosis of cancer. The association remained highly significant for both 8-oxodG and 8-oxoGuo, in both linear (*P*<0.001 for both markers) and multivariate regression analysis (*P* = 0.002 and *P*<0.001, respectively). To further characterise the significance of the association, we plotted median 8-oxodG and 8-oxoGuo in relation to quartiles of cortisol excretion. Individuals in the lowest cortisol excretion quartile had substantially lower excretion of DNA and RNA oxidation markers compared to individuals in the highest quartile (8-oxodG: median = 12.49 [interquartile range: 9.03–14.97] vs 19.65 [14.23–24.55] nmol/24 h, respectively. 8-oxoGuo: 20.23 [15.40–25.33] vs 32.64 [25.23–40.38] nmol/24 h, respectively) (Figure 1B).

**Table 1:** Demographical, clinical and biochemical characteristics of the cohort.

**Medical history<\emph>**	
Previous diagnosis of cancer	22/211 (10%)
Cancer diagnosis last 10 years	8/211 (4%)
Diabetes	11/209 (5%)
Chronic bronchitis	2/209 (1%)
Bronchial asthma	0/209 (0%)
Emphysema	5/209 (2%)
CHF	6/209 (3%)
Parkinson's disease	2/209 (1%)
Hypertension	19/208 (9%)
Angina pectoris	8/209 (4%)
Stroke	6/209 (3%)
Myocardial infarction	4/209 (2%)
Chronic liver disease	2/209 (1%)
**Clinical characteristics<\emph>**	
BMI (kg/m^2^)	26.9 (24.5–29.6)
Smoking	
Smoker	41 (20%)
Non-smoker	166 (80%)
Demographic characteristics	
Age	72 (69–76)
Male sex	108 (51%)
**Laboratory variables<\emph>**	
Serum creatinine (mg/dL)	0.9 (0.8–1.0)
Cholesterol total (mg/dL)	221.0±38.4
Triglycerides (mg/dL)	109 (81–151)
HS-CRP (µg/mL)	2.20 (0.97–8.62)
IL-6 (pg/mL)	1.03 (0.63–3.23)
Systolic blood pressure (mmHg)	150 (130–160)
Diastolic blood pressure (mmHg)	80 (80–90)

Footnote: The total number of subjects in each disease category varies due to missing data. Laboratory variables are given as median (interquartile range) or mean ± standard deviation.

**Table 2:** Multivariate regression analysis.

	log 8-oxodG					log 8-oxoGuo				
	*β*	SE	Partial *R^2^*	*P*	Adjusted *R^2^*	*β*	SE	Partial *R^2^*	*P*	Adjusted *R^2^*
Model				<0.0001	0.3041				<0.0001	0.2156
Cortisol	0.0031	0.0007	0.1067	<0.0001		0.0026	0.0007	0.0945	<0.0001	
Ferritin	0.0013	0.0002	0.1950	<0.0001		0.0009	0.0002	0.1086	<0.0001	
Age	−0.0068	0.0065	0.0064	0.2983		0.0008	0.0062	0.0001	0.9824	
Sex	0.0308	0.0650	0.0064	0.6357		−0.0076	0.0629	<0.0001	0.9041	
Body Mass Index	−0.0033	0.0068	0.0014	0.6255		0.0067	0.0064	0.0070	0.4256	
Glucose	−0.0006	0.0013	0.0015	0.6111		0.0022	0.0012	0.0236	0.0562	
Insulin	0.0019	0.0050	0.0009	0.7011		0.0061	0.0051	0.0093	0.2328	
Inflammation status^a^	−0.0790	0.0585	0.0108	0.1785		−0.0031	0.0558	<0.0001	0.9558	
Smoking status^b^	−0.0701	0.0634	0.0072	0.2700		−0.0489	0.0612	0.0042	0.4256	
Cancer status^c^	0.1727	0.0980	0.0182	0.0799		−0.0334	0.0937	0.0008	0.7221	

Footnote: *β* = regression coefficient. SE  =  standard error. *R^2^* =  coefficient of determination. ^a^ “Low-grade inflammation” or “no inflammation”. ^b^ Smoker or non-smoker. ^c^ ± previous diagnosis of cancer.

## Discussion

The result has implications for the accelerated aging and risk for disease associated with psychological stress states [6]. Depression, which is very often associated with hypercortisolism [17], has an increased non-suicide mortality, comparable in size to the excess mortality associated with smoking [5], [18]. Perceived psychological stress in mothers giving care to chronically ill children was associated with shortened telomeres, which is a proposed measure of biological age [19]. Similar results have been obtained in adult persons with childhood adversities as well as in anxiety, mood disorder, and schizophrenia patients [20]–[22].

It has been suggested that these pro-aging effects of stress are mediated by hormonal changes, in particular the prolonged elevation of cortisol [6]. The cardiovascular comorbidity of stress and depression is high [2], [5], and hypercortisolemic depression has been shown to be associated with the metabolic syndrome [23]. Furthermore, it was recently demonstrated that higher urinary cortisol excretion in itself is a predictor of cardiovascular mortality [24]. However, it should be noted that not all psychological stress is associated with hypercortisolism. Stress states such as PTSD have been associated with hypocortisolism, and this has even been hypothesized to have protective somatic effects [25].

Here we provide evidence for a robust association between cortisol excretion and the amount of oxidatively induced damage to DNA and RNA. The increase in DNA/RNA oxidation marker excretion with increased cortisol excretion (i.e. being in either the upper vs. the lower quartile of cortisol excretion) is comparable to, if not exceeding, the increase observed in smoking [26], a leading age-advancing factor [27].

Genotoxic stress such as DNA damage is suspected to be a major contributor to the aging process [7]. The oxidatively generated damage to DNA accumulates with age and can cause single and double strand breaks, which may lead to cell senescence, mutagenesis or apoptosis [8]. Animals and humans with genetic defects in nuclear DNA repair phenotypically display premature aging [28]. Oxidatively induced damage to telomeres affects telomeric integrity and reduces telomere length [29], [30]. Therefore, an association between cortisol and 8-oxodG/8-oxoGuo could be of importance to the link between stress, aging and age-related disease. Since genomic damage may cause mutations that can lead to cancer, it could also be expected from this association that stress increases the risk for malignant disease. However, while stress might have a promoting effect on existing cancers, there is no clinical evidence to suggest that stress increase cancer risk *per se* [4].

There are several possible mechanisms that could account for the presented association. Cortisol is released from the adrenal glands by activation of the hypothalamic-pituitary-adrenal (HPA) axis in response to psychological stress, as well as to a variety of physiological challenges. The hormone binds to intracellular glucocorticoid receptors, and exerts its effects by altering gene expression [31]. Hence, one could speculate that oxidative stress effects are mediated by transcriptionally determined changes in DNA/RNA repair, antioxidant defence systems and/or ROS formation. In fact, in an *in vitro* study using murine fibroblasts, corticosteroids (as well as catecholamines) were shown to induce DNA damage (as measured by the comet assay), decrease DNA repair and modulate the expression of several genes involved in DNA damage responses [32]. Corticosteroid treatment and chronic “restraint stress” have been shown to reduce antioxidant defenses in the brain, liver and heart of rats [11]. Finally, long-term corticosteroid treatment negatively influences various measures of mitochondrial function (oxidation, membrane potential and calcium holding capacity) [33], and thus could also be suspected to induce increased leakage of ROS during mitochondrial respiration, although this has never, to our knowledge, been directly investigated.

The net result of these diverse effects is likely to be a general increase in oxidative damage to DNA and RNA. The abovementioned finding of a dose-dependent increase in hepatic mitochondrial DNA oxidation after corticosterone treatment is in line with this notion [12]. Experimental induction of oxidative DNA damage by an exogenous carcinogen leads to increased urinary excretion of 8-oxodG [34]. Hence, a general increase in whole-body nucleic acid oxidation caused by higher cortisol levels would most likely also entail increased urinary excretion of DNA/RNA oxidation markers.

The level of cortisol excretion was correlated with both DNA and RNA oxidation. DNA is stable and primarily present in the nucleus of cells, whereas RNA has a rapid turn-over and is present primarily outside the nucleus. The two nucleic acid oxidation markers were highly correlated [16]. This would indicate that high cortisol can lead to oxidative modifications of different molecules in different parts of cells, and thus a general state of oxidative stress rather than DNA or RNA oxidation specifically. Accumulating evidence suggest that RNA damage may play an important role in human pathophysiology by interfering with mRNA translation and thereby protein expression [35], [36].

It should be firmly emphasized, however, that the present results are correlational, and therefore do not allow for any conclusions on the biological events that link cortisol and 8-oxodG/8-oxoGuo excretion to each other. Cortisol secretion might be associated with other stress-related hormonal changes, which can also lead to oxidative stress (e.g catecholamines). Furthermore, it is possible that individuals with high oxidative stress due to other conditions concomitantly have an increased HPA-axis activity. However, the fact that the cohort was generally healthy, and that the analysis was robust for the adjustment for multiple known or possible confounders of oxidative stress, indicates that this is not the case in our study. Our findings are based on a cross-sectional investigation of a cohort of elderly individuals, and cannot necessarily be extended to younger persons. HPA-axis activity is exaggerated in the elderly [37], and antioxidant defences may be impaired [38]. This combination entails that the association between cortisol and oxidative stress could be stronger – and thus perhaps more readily uncovered - in the elderly than in younger individuals.

It is not possible to establish the anatomical origin of the urinary markers, and importantly, some tissues may contribute more than others. The brain, for example, has a high glucocorticoid sensitivity, a large oxygen consumption per mass tissue and relatively modest antioxidant defence [31], [39], and therefore may be particularly prone to cortisol-induced oxidative stress. This specific vulnerability of the brain is supported by evidence suggesting an important role of oxidative stress in the pathogenesis of neurodegenerative disorders, including Alzheimer's disease [39], [40]. In that context, it is notable that depression appears to increase the risk of dementia in a dose-response manner (i.e. risk increasing with the number of depressive episodes) [41], [42].

The urinary excretion of oxidized nucleosides is widely considered to reflect the rate of whole body DNA/RNA oxidation. That said, the exact origins of these urinary markers are not determined, and several repair enzymes have been implicated [43]. In principle, these could be differentially regulated by cortisol, influencing the formation of free oxidized nucleosides in unpredictable ways. However, we would argue that in most in vivo situations, enzymatic repair activity will follow first order kinetics and thereby the amount of oxidation, meaning that inter- or intraindividual differences in urinary excretion of 8-oxodG/8-oxoGuo do in fact reflect differences in oxidative stress on DNA/RNA, rather than differences in repair activity [44].

The study was based on a subsample of the InChianti cohort, which was originally selected for another study, namely the association between low-grade inflammation and oxidative nucleic acid oxidation (see “Methods”). Somewhat surprisingly, we did not find evidence to support such an association. This has been discussed elsewhere [16]. There was no difference in either cortisol or 8-oxodG/8-oxoGuo excretion in the low-grade vs no inflammation group, and no difference in cortisol excretion between the subsample and the entire cohort. We can not rule out that differences in cellular glucocorticoid sensitivity or diurnal cortisol rythm, which is not detected by the 24 h excretion of cortisol, could exist between the inflammation groups. Gene expression studies using peripheral blood monocytes has suggested that stress associated with caregiving may reduce cellular glucocorticoid sensitivity, and increase the expression of pro-inflammatory factors such as NF-kβ, in the absence of increased salivary cortisol secretion [45]. However, such potential differences in our groups have not resulted in differing 8-oxodG/8-oxoGuo excretion. Furthermore, there was no effect on the result when adjusting for inflammation status. Based on this, we consider the subsample to be applicable for the present study.

In conclusion, we report an association between 24 h urinary excretion of cortisol and markers of oxidatively generated DNA and RNA damage in a population of elderly individuals, which persisted after the adjustement for multiple confounders of oxidative stress. Although causality cannot be inferred from this cross-sectional study, the suggestion of a causal relationship is strengtened by experimental interventions showing induction of oxidatively generated DNA damage by corticosterone *in vivo* [12], as well as by plausible biological mechanisms demonstrated *in vitro* [32], [33]. The finding adds support to the hypothesis that cortisol-induced damage to DNA/RNA is an explanatory factor in the complex relation between stress, aging and disease. Further studies on the association are needed, e.g. animal and *in vitro* studies, which would allow for interventions that could shed more light on the potential causal mechanisms involved.

## Materials and Methods

### Ethics Statement

The Italian National Institute of Research and Care on Aging Ethical Committee approved the study protocol, which complied with the principles stated in the Declaration of Helsinki.

### Cohort

InCHIANTI is an epidemiological study of risk factors contributing to the declining ability to walk in late life. The study was performed in two small towns located in Tuscany (Italy): Greve in Chianti and Bagno a Ripoli, between September 1998 and April 2000. The design and data collection methods of InCHIANTI are described in detail elsewhere [46]. The study population consisted of a random sample of the population aged 65 years and older selected from the city registry of the two municipalities, and an additional small sample of younger people. Of the 1530 subjects originally sampled, 1453 agreed to participate in the study. Participants received an extensive description of the study and participated after providing written informed consent.

The subsample (n = 220) was initially selected from the upper and lower tertiles of selected markers of inflammation (CRP and IL-6), to study whether low-grade inflammation was associated with increased nucleic acid oxidation [16]. The subsample included subjects between 65 and 83 years of age. 110 “low-grade inflammation” cases were selected from the top tertile of both serum CRP (median  = 8.62 [interquartile range  = 5.68 - 14.10] µg/ml) and IL-6 (3.22 [2.31–4.88] pg/ml) and were matched by age and sex with 110 “no-inflammation” controls found in the lowest tertile of CRP and IL-6 (0.97 [0.62–1.27] µg/ml and 0.63 [0.46–0.82] pg/ml, respectively). In nine subjects the remaining urine volume was insufficient for analysis. There was no difference in urinary cortisol excretion between cases and controls (98.2 [68.1–124.7] µg and 104.6 [72.9–126.0] µg respectively, *p* = 0.48). There was no difference between urinary cortisol excretion in the subsample vs the entire cohort (100.8 [70.0 – 125.8] µg and 95.3 [70.3 – 124.3] µg respectively, *p* = 0.52).

### Determination of cortisol, 8-oxodG and 8-oxoGuo

Before an in-clinic assessment, study participants collected all urine produced during a 24 h period starting after the first voided urine following awakening and including the first voided urine on the following day. At assessment, 10 ml aliquots of urine were prepared and stored at –80°C for later assaying at the Clinical Chemistry Laboratory of the Careggi Hospital, Italy. Completeness of urine collection was evaluated using 24-h urinary creatinine excretion. Subjects with urinary creatinine level <6 mmol/day plus total urine volume <1000 L/day or with a urinary creatinine level <5 mmol/day were identified as having incomplete urine collection [47]. Urinary cortisol was measured by an immunochemiluminescence method and an ADVIA-Centaur immunoassay system (Bayer Diagnostics). The intra-assay coefficient of variation was less than 2.0% and the inter-assay coefficient of variation was less than 3.0%. Urinary cortisol excretion was defined as µg of cortisol excreted over 24 h [23].

The same samples were assayed for the oxidatively modified nucleosides 8-oxodG and 8-oxoGuo using high-performance liquid chromatography-tandem mass spectrometry (HPLC MS/MS) at the Department of Clinical Pharmacology, Rigshospitalet, Denmark. Urinary 8-oxodG is stable for at least 15 years when stored at -20 degrees celcius [48]. Chromatographic separation was performed on a Perkin Elmer Series 200 HPLC equipped with two pumps, autosampler, solvent cabinet and vacuum degasser (Perkin Elmer, Norwalk, CT, United States). The column used was a Phenomenex Prodigy ODS HPLC column (100×2 mm, 3 µ) protected with a C18 (ODS) guard column (4×2 mm), both obtained from Phenomenex (Torrance, CA, United States). The mass spectrometric detection was performed on an API 3000 triple quadrupole mass spectrometer (Sciex, Toronto, Canada) equipped with a turboionspray source (Turbospray). Details of the analysis are described elsewhere [15]. Urinary excretion of 8-oxodG and 8-oxoGuo was defined as nmol excreted over 24 h.

### Statistics

Simple and multivariate linear regression analyses were used to determine the association between urinary cortisol excretion and urinary 8-oxodG and 8-oxoGuo excretion. One extreme outlier was omitted (cortisol value both outside the 99^th^ percentile of the data and above the upper normal limit for 24 h cortisol excretion of 350 nmol/24 h). Due to deviation from normal distribution the variables 8-oxodG and 8-oxoGuo were log-transformed before calculation, which resulted in normal distribution of both variables. To determine differences between the subsample and the entire InChianti cohort, and between the high and low inflammation groups in the subsample, variables were tested using Wilcoxon signed-rank test. All statistical analyses were performed using the SAS software version 9.1 (SAS Institute Inc. Cary, NC, USA). Statistical significance was defined as *P*<0.05. All statistical tests were two sided.

## References

[1] McEwen BS (1998). Protective and damaging effects of stress mediators.. N Engl J Med.

[2] Brotman DJ, Golden SH, Wittstein IS (2007). The cardiovascular toll of stress.. Lancet.

[3] Sapolsky RM (1996). Why stress is bad for your brain.. Science.

[4] Reiche EM, Nunes SO, Morimoto HK (2004). Stress, depression, the immune system, and cancer.. Lancet Oncol.

[5] Wulsin LR, Evans JC, Vasan RS, Murabito JM, Kelly-Hayes M (2005). Depressive symptoms, coronary heart disease, and overall mortality in the Framingham Heart Study.. Psychosom Med.

[6] Epel ES (2009). Psychological and metabolic stress: a recipe for accelerated cellular aging?. Hormones (Athens ).

[7] Finkel T, Holbrook NJ (2000). Oxidants, oxidative stress and the biology of ageing.. Nature.

[8] Maynard S, Schurman SH, Harboe C, de Souza-Pinto NC, Bohr VA (2009). Base excision repair of oxidative DNA damage and association with cancer and aging.. Carcinogenesis.

[9] Liu J, Wang X, Shigenaga MK, Yeo HC, Mori A (1996). Immobilization stress causes oxidative damage to lipid, protein, and DNA in the brain of rats.. FASEB J.

[10] Song L, Zheng J, Li H, Jia N, Suo Z (2009). Prenatal stress causes oxidative damage to mitochondrial DNA in hippocampus of offspring rats.. Neurochem Res.

[11] Zafir A, Banu N (2008). Modulation of in vivo oxidative status by exogenous corticosterone and restraint stress in rats.. Stress.

[12] Caro P, Gomez J, Sanz A, Portero-Otin M, Pamplona R (2007). Effect of graded corticosterone treatment on aging-related markers of oxidative stress in rat liver mitochondria.. Biogerontology.

[13] Irie M, Tamae K, Iwamoto-Tanaka N, Kasai H (2005). Occupational and lifestyle factors and urinary 8-hydroxydeoxyguanosine.. Cancer Sci.

[14] Forlenza MJ, Miller GE (2006). Increased serum levels of 8-hydroxy-2′-deoxyguanosine in clinical depression.. Psychosom Med.

[15] Weimann A, Belling D, Poulsen HE (2002). Quantification of 8-oxo-guanine and guanine as the nucleobase, nucleoside and deoxynucleoside forms in human urine by high-performance liquid chromatography-electrospray tandem mass spectrometry.. Nucleic Acids Res.

[16] Broedbaek K, Siersma V, Andersen JT, Petersen M, Afzal S (2011). The association between low-grade inflammation, iron status and nucleic acid oxidation in the elderly..

[17] Holsboer F (2000). The corticosteroid receptor hypothesis of depression.. Neuropsychopharmacology.

[18] Mykletun A, Bjerkeset O, Overland S, Prince M, Dewey M (2009). Levels of anxiety and depression as predictors of mortality: the HUNT study.. Br J Psychiatry.

[19] Epel ES, Blackburn EH, Lin J, Dhabhar FS, Adler NE (2004). Accelerated telomere shortening in response to life stress.. Proc Natl Acad Sci U S A.

[20] Simon NM, Smoller JW, McNamara KL, Maser RS, Zalta AK (2006). Telomere shortening and mood disorders: preliminary support for a chronic stress model of accelerated aging.. Biol Psychiatry.

[21] Kao HT, Cawthon RM, Delisi LE, Bertisch HC, Ji F (2008). Rapid telomere erosion in schizophrenia.. Mol Psychiatry.

[22] Kananen L, Surakka I, Pirkola S, Suvisaari J, Lonnqvist J (2010). Childhood adversities are associated with shorter telomere length at adult age both in individuals with an anxiety disorder and controls.. PLoS One.

[23] Vogelzangs N, Suthers K, Ferrucci L, Simonsick EM, Ble A (2007). Hypercortisolemic depression is associated with the metabolic syndrome in late-life.. Psychoneuroendocrinology.

[24] Vogelzangs N, Beekman AT, Milaneschi Y, Bandinelli S, Ferrucci L (2010). Urinary cortisol and six-year risk of all-cause and cardiovascular mortality.. J Clin Endocrinol Metab.

[25] Fries E, Hesse J, Hellhammer J, Hellhammer DH (2005). A new view on hypocortisolism.. Psychoneuroendocrinology.

[26] Prieme H, Loft S, Klarlund M, Gronbaek K, Tonnesen P (1998). Effect of smoking cessation on oxidative DNA modification estimated by 8-oxo-7,8-dihydro-2′-deoxyguanosine excretion.. Carcinogenesis.

[27] Vita AJ, Terry RB, Hubert HB, Fries JF (1998). Aging, health risks, and cumulative disability.. N Engl J Med.

[28] Lombard DB, Chua KF, Mostoslavsky R, Franco S, Gostissa M (2005). DNA repair, genome stability, and aging.. Cell.

[29] Wang Z, Rhee DB, Lu J, Bohr CT, Zhou F (2010). Characterization of oxidative guanine damage and repair in mammalian telomeres.. PLoS Genet.

[30] Richter T, von Zglinicki T (2007). A continuous correlation between oxidative stress and telomere shortening in fibroblasts.. Exp Gerontol.

[31] Chrousos GP, Kino T (2009). Glucocorticoid signaling in the cell. Expanding clinical implications to complex human behavioral and somatic disorders.. Ann N Y Acad Sci.

[32] Flint MS, Baum A, Chambers WH, Jenkins FJ (2007). Induction of DNA damage, alteration of DNA repair and transcriptional activation by stress hormones.. Psychoneuroendocrinology.

[33] Du J, Wang Y, Hunter R, Wei Y, Blumenthal R (2009). Dynamic regulation of mitochondrial function by glucocorticoids.. Proc Natl Acad Sci U S A.

[34] Deng XS, Tuo J, Poulsen HE, Loft S (1998). Prevention of oxidative DNA damage in rats by brussels sprouts.. Free Radic Res.

[35] Shan X, Chang Y, Lin CL (2007). Messenger RNA oxidation is an early event preceding cell death and causes reduced protein expression.. FASEB J.

[36] Tanaka M, Chock PB, Stadtman ER (2007). Oxidized messenger RNA induces translation errors.. Proc Natl Acad Sci U S A.

[37] Otte C, Hart S, Neylan TC, Marmar CR, Yaffe K (2005). A meta-analysis of cortisol response to challenge in human aging: importance of gender.. Psychoneuroendocrinology.

[38] Sfar S, Jawed A, Braham H, Amor S, Laporte F (2009). Zinc, copper and antioxidant enzyme activities in healthy elderly Tunisian subjects.. Exp Gerontol.

[39] Halliwell B (2006). Oxidative stress and neurodegeneration: where are we now?. J Neurochem.

[40] Lovell MA, Markesbery WR (2001). Ratio of 8-hydroxyguanine in intact DNA to free 8-hydroxyguanine is increased in Alzheimer disease ventricular cerebrospinal fluid.. Arch Neurol.

[41] Kessing LV, Andersen PK (2004). Does the risk of developing dementia increase with the number of episodes in patients with depressive disorder and in patients with bipolar disorder?. J Neurol Neurosurg Psychiatry.

[42] Saczynski JS, Beiser A, Seshadri S, Auerbach S, Wolf PA (2010). Depressive symptoms and risk of dementia: the Framingham Heart Study.. Neurology.

[43] Evans MD, Saparbaev M, Cooke MS (2010). DNA repair and the origins of urinary oxidized 2′-deoxyribonucleosides.. Mutagenesis.

[44] Poulsen HE (2005). Oxidative DNA modifications.. Exp Toxicol Pathol.

[45] Miller GE, Chen E, Sze J, Marin T, Arevalo JM (2008). A functional genomic fingerprint of chronic stress in humans: blunted glucocorticoid and increased NF-kappaB signaling.. Biol Psychiatry.

[46] Ferrucci L, Bandinelli S, Benvenuti E, Di IA, Macchi C (2000). Subsystems contributing to the decline in ability to walk: bridging the gap between epidemiology and geriatric practice in the InCHIANTI study.. J Am Geriatr Soc.

[47] Murakami K, Sasaki S, Takahashi Y, Uenishi K, Watanabe T (2008). Sensitivity and specificity of published strategies using urinary creatinine to identify incomplete 24-h urine collection.. Nutrition.

[48] Loft S, Svoboda P, Kasai H, Tjonneland A, Vogel U (2006). Prospective study of 8-oxo-7,8-dihydro-2′-deoxyguanosine excretion and the risk of lung cancer.. Carcinogenesis.

